# Increased Sleep Depth in Developing Neural Networks: New Insights from Sleep Restriction in Children

**DOI:** 10.3389/fnhum.2016.00456

**Published:** 2016-09-21

**Authors:** Salome Kurth, Douglas C. Dean, Peter Achermann, Jonathan O’Muircheartaigh, Reto Huber, Sean C. L. Deoni, Monique K. LeBourgeois

**Affiliations:** ^1^Sleep and Development Laboratory, Department of Integrative Physiology, University of Colorado Boulder, BoulderCO, USA; ^2^Pulmonary Clinic, Division of Pulmonology, University Hospital ZurichZurich, Switzerland; ^3^Advanced Baby Imaging Laboratory, School of Engineering, Brown University, ProvidenceRI, USA; ^4^Waisman Laboratory for Brain Imaging and Behavior, University of Wisconsin-Madison, MadisonWI, USA; ^5^Chronobiology and Sleep Research, Institute of Pharmacology and Toxicology, University of ZurichZurich, Switzerland; ^6^Centre for the Developing Brain, Division of Imaging Sciences & Biomedical Engineering, King’s College LondonLondon, UK; ^7^Department of Neuroimaging, Institute of Psychiatry, Psychology & Neuroscience, King’s College LondonLondon, UK; ^8^Child Development Center, University Children’s Hospital ZurichZurich, Switzerland; ^9^Department of Child and Adolescent Psychiatry and Psychotherapy, Psychiatric Hospital University of ZurichZurich, Switzerland; ^10^Children’s Hospital Colorado, School of Medicine, University of Colorado, AuroraCO, USA

**Keywords:** brain development, myelin, sleep deprivation, sleep EEG, high density EEG, slow wave activity, brain maturation, mcDESPOT

## Abstract

Brain networks respond to sleep deprivation or restriction with increased sleep depth, which is quantified as slow-wave activity (SWA) in the sleep electroencephalogram (EEG). When adults are sleep deprived, this homeostatic response is most pronounced over prefrontal brain regions. However, it is unknown how children’s developing brain networks respond to acute sleep restriction, and whether this response is linked to myelination, an ongoing process in childhood that is critical for brain development and cortical integration. We implemented a bedtime delay protocol in 5- to 12-year-old children to obtain partial sleep restriction (1-night; 50% of their habitual sleep). High-density sleep EEG was assessed during habitual and restricted sleep and brain myelin content was obtained using mcDESPOT magnetic resonance imaging. The effect of sleep restriction was analyzed using statistical non-parametric mapping with supra-threshold cluster analysis. We observed a localized homeostatic SWA response following sleep restriction in a specific parieto-occipital region. The restricted/habitual SWA ratio was negatively associated with myelin water fraction in the optic radiation, a developing fiber bundle. This relationship occurred bilaterally over parieto-temporal areas and was adjacent to, but did not overlap with the parieto-occipital region showing the most pronounced homeostatic SWA response. These results provide evidence for increased sleep need in posterior neural networks in children. Sleep need in parieto-temporal areas is related to myelin content, yet it remains speculative whether age-related myelin growth drives the fading of the posterior homeostatic SWA response during the transition to adulthood. Whether chronic insufficient sleep in the sensitive period of early life alters the anatomical generators of deep sleep slow-waves is an important unanswered question.

## Introduction

One fundamental question in the field of neurodevelopment concerns how inadequate sleep in children affects brain activity and associated cognitive and behavioral outcomes (Paus et al., 2008). A growing epidemiological literature indicates that childhood sleep disturbance predicts future cognitive, attentional, and psychosocial problems (Gregory et al., 2005; Simola et al., 2014). Animal studies show that during critical periods of life, sleep is required for plastic processes underlying the maturation of specific brain circuits (Jha et al., 2005; Kayser et al., 2014). In adult humans, extending wakefulness is an experimental approach for investigating the effects of inadequate sleep on the brain. Yet, to date, it is unknown which brain circuits are sensitive to restricted sleep (RS) in children.

The state of deep sleep is associated with changes in neuronal network organization (Vyazovskiy et al., 2009; Boly et al., 2012; Kurth et al., 2013). In adults, extending wakefulness triggers a homeostatic response in subsequent recovery sleep that is best quantified as increased slow-wave activity (SWA, 1–4.5 Hz) of the sleep electroencephalogram (EEG; Achermann and Borbély, 2011). SWA reflects a recovery process in cortical and subcortical structures that arises from highly synchronized neuronal activity (Vyazovskiy et al., 2009) and reliably reflects the depth of sleep (Achermann and Borbély, 2011), as well as local brain plasticity (Huber et al., 2004, 2006). Learning or sensory stimulation during wakefulness locally enhances SWA in subsequent sleep (Kattler et al., 1994; Huber et al., 2004; Wilhelm et al., 2014), suggesting a local and use-dependent regulation of SWA.

In adults, SWA is most predominant in frontal brain regions, which are the same areas that show the largest SWA increase following sleep deprivation (Cajochen et al., 1999; Finelli et al., 2001). Frontal regions sustain a broad range of cognitive demands (Duncan and Owen, 2000) and are likely most plastic when neuronal networks are fully mature. Functions related to the prefrontal cortex (e.g., working-memory, attention, arithmetic performance, word fluency) are impaired by sleep deprivation (Harrison and Horne, 1998; Drummond et al., 1999; Chee and Choo, 2004). One prominent difference between children and adults is the local predominance of SWA: a posterior-to-anterior shift is observed across the first two decades of life, a spatial trajectory that is consistent with the development of cortical anatomy (Kurth et al., 2010, 2012). Evidence is converging that SWA topography reflects developing networks (Kurth et al., 2010, 2012; Buchmann et al., 2011b) and their altered trajectories in clinical groups (Ringli et al., 2013; Tesler et al., 2013; Bolsterli Heinzle et al., 2014).

We investigated school-age children’s neuronal response to acute sleep restriction by examining the effects of extending wakefulness on cortical brain activity during sleep. Further, we addressed whether the neuronal recovery response during sleep is correlated with underlying white matter myelination, a cornerstone of brain development. Because children at this developmental stage exhibit equal predominance of SWA in posterior and prefrontal areas (Kurth et al., 2010), we hypothesized a homeostatic SWA response to acute sleep restriction over both regions. As the development of cortical and subcortical networks are linked (Kubota et al., 2013; Alcauter et al., 2014), we also explored associations between children’s SWA response to acute sleep restriction and subcortical local myelin content.

## Materials and Methods

**FIGURE 1:**
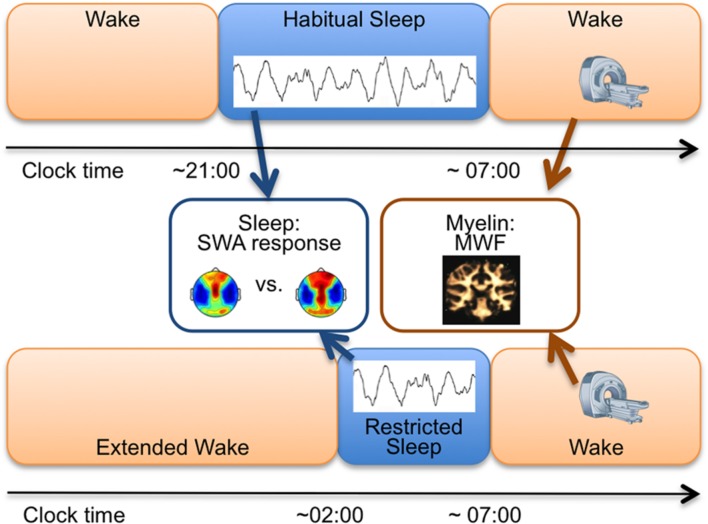
**Study Protocol.** Study measures for each subject involved: (i) One night of high-density EEG during habitual sleep (HS) and restricted sleep (RS; blue boxes) to assess slow-wave activity (SWA, 1–4.5 Hz) topography and to compare SWA between conditions and (ii) mcDESPOT magnetic resonance imaging scan in the morning either in the HS or the RS condition to assess myelin water fraction (MWF) masks. The two conditions were counterbalanced and separated by at least 7 days.

Healthy children (*n* = 13; 5–12 years; six females) followed an individualized, strict sleep/wakefulness schedule (verified with actigraphy and sleep diaries) to stabilize sleep at least 5 days before each of two counterbalanced assessments (**Figure 1**). One night of habitual sleep (HS) was scheduled according to reported habitual bedtimes, i.e., stabilized sleep times. The RS condition involved an acute 50% reduced bedtime achieved via a bedtime delay (1-night), while maintaining the same morning wake time. Average bedtime was 20:58 ± 0:08 (M ± SE, clock time) for HS and 2:04 ± 0:03 for RS. Average wake time was 07:00 ± 0:01 for HS and 06:59 ± 0:05 for RS. To achieve sleep restriction, children were kept awake by interacting with the research team in games or reading. At-home all-night 128-channel high-density EEG (Electrical Geodesics, Inc., Sensor Net, portable system) was obtained for HS and RS. Multicomponent relaxometry mcDESPOT (multi-component driven equilibrium single pulse observation of T_1_ and T_2_) MRI (Deoni et al., 2013) was performed in all subjects to quantify individual maps of myelin water fraction (MWF; **Figure 1**). Before enrollment and after explanation of the study protocol, methods and aims, written parental consent and child assent was obtained. The Institutional Review Board at Brown University approved all procedures, and the study was performed according to the Declaration of Helsinki.

### Sleep EEG

**FIGURE 2:**
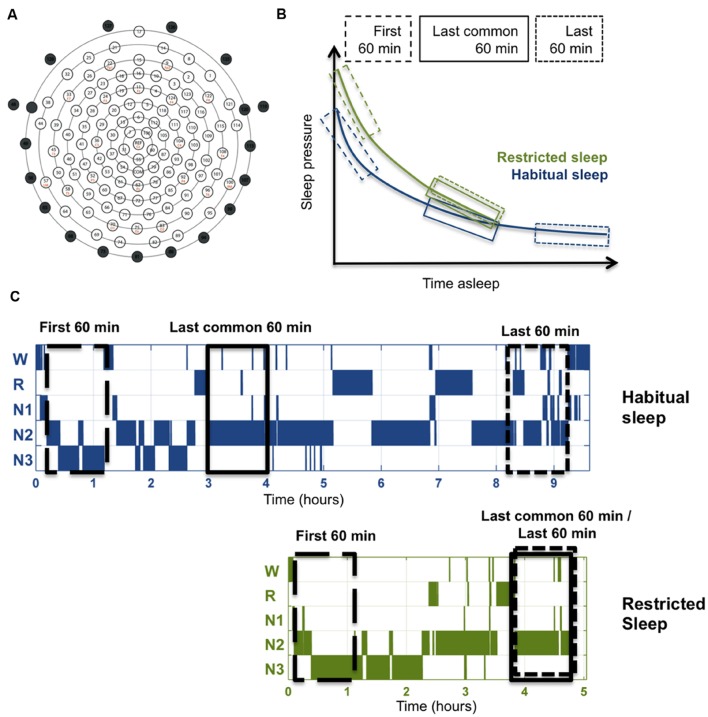
**Method illustrations.** **(A)** Layout of high-density EEG electrode net in top view (adapted from Electrical Geodesics, Inc.). Marked electrodes (numbered 43, 48, 49, 56, 63, 68, 73, 81, 88, 94, 99, 107, 113, 119, 120, 125, 126, 127, and 128) were excluded (marked as black circles). The remaining 109 electrodes were included in the analysis. **(B)** Illustration of the time windows used for the three different comparisons in relation to the homeostatic decrease of sleep pressure. Each time window included 60 min artifact-free non-REM sleep (stages N2 and N3), and was identified for each individual separately. The time point of the last common 60 min in HS was determined according to minutes of non-REM sleep passed to the corresponding 60-min-window in RS. **(C)** Illustration of the three time windows exemplified in typical hypnograms for the two sleep conditions.

Electrode nets were adjusted to vertex and mastoids and filled with gel electrolyte (ECI, Electro Gel). Following standard laboratory procedures (Kurth et al., 2010), i.e., impedances were below 50 kΩ at the start of the recording, the EEG was sampled at 500 Hz, filtered from 0.01 to 200 Hz and referenced to the vertex. Data were filtered offline (bandpass 0.5–50 Hz) and down-sampled to 128 Hz. Data were then re-referenced to the average across all channels, and standard sleep scoring (Iber et al., 2007) was performed. Artifacts were rejected semi-automatically on a 20-s basis (visual inspection and power exceeding a threshold based on average power in 0.75–4.5 and 20–30 Hz), and poor quality channels were excluded. Power spectral analysis was performed for all EEG derivations as previously published (Kurth et al., 2010), including Fast Fourier transform routine, 20-s-epochs (averages of five 4-s epochs; Hanning window; no overlap; using pwelch from the signal processing toolbox, MATLAB, MathWorks) resulting in a frequency resolution of 0.25 Hz. The 20-s spectral power values of the artifact-free 20-s-epochs were then averaged to determine SWA in the reported time windows (first 60 min, last 60 min and last common 60 min, i.e., 180 20-s epochs for each time window). Epochs containing artifacts were skipped, such that the same number of epochs was met in each subject. We used the topoplot function of the EEGLab Matlab toolbox to display EEG activity (Delorme and Makeig, 2004). According to a standard approach, values of 109 channels “above the ears” were included, in order to minimize artifacts from facial or neck muscles [as commonly performed, e.g., (Ferrarelli et al., 2007), **Figure 2A**]. Three different time windows were utilized in this analysis (**Figures 2B,C**): (i) the first 60 min – to examine the effects of RS under the highest level of sleep pressure; (ii) the last 60 min – to compare the effects of RS at the end of the sleep episode, just before awakening; and (iii) the last common 60 min – to examine sleep restriction effects during a late window in the sleep episode, while controlling for the homeostatic decrease of sleep pressure, i.e., after a similar duration of sleep. The 60 min-windows of artifact-free non-REM sleep (stages N2 and N3) and data-driven clusters of electrodes were analyzed. Statistical non-parametric mapping (SnPM) was performed using a supra-threshold cluster analysis, with single threshold tests representing an increased or more restrictive level of significance (Nichols and Holmes, 2002), which controls for multiple comparisons (Huber et al., 2004; Ferrarelli et al., 2007; Aepli et al., 2015). *T*-values were calculated for each electrode; the maximal *t*-value across all electrodes was identified in all permutations. From this distribution, the 95th percentile was defined as the critical value for single threshold tests. Neighboring electrodes exceeding a *t*-value of 2.18 (corresponding to *p* = 0.05 with *n* = 13) were considered a cluster. In short, data (8192, i.e., 2ˆ13) were generated from original data by randomly assigning the condition label and paired *t*-test were performed. The maximal size of resulting electrode clusters from these new datasets that exceeded a *t*-value of 2.18 was counted, and the 95th percentile cluster size was determined. For the HS vs. RS comparison, only clusters above this critical cluster size were considered significant (Nichols and Holmes, 2002; Huber et al., 2004). Whether electrodes showing a significant effect were neighbored and belonged to the same cluster was determined using electrode locations provided in the fieldtrip toolbox (Matlab). SnPM was also used to compare anatomical MWF masks (see details below) with the sleep-restriction induced changes in the EEG. Pearson correlations were performed at each derivation. To control for multiple comparisons of correlations, 20,000 permutations were executed, with randomization of the order of the subjects and a critical value *r* = 0.55 (corresponding to *p* = 0.05, two-tailed with *n* = 13; Nichols and Holmes, 2002).

### mcDESPOT Imaging

All children were imaged using a 3T Siemens Trio scanner, equipped with a 12-channel head RF array while watching a movie. Age-appropriate mcDESPOT protocols comprise a series of spoiled gradient recalled echo (SPGR) images and fully balanced steady-state free precession (bSSFP) images acquired over a range of flip angles (Deoni et al., 2012). Inversion-prepared (IR-) SPGR data were acquired to correct for transmit magnetic field (B_1_) inhomogeneities, and the bSSFP data were acquired with two different phase-cycling patterns to allow correction for main magnetic field (B_0_) inhomogeneities. A constant voxel dimension (1.8 mm × 1.8 mm × 1.8 mm) was used, with the field of view and imaging matrix adjusted for age and head size. To reduce acoustic noise, the maximum imaging gradient slew rates and peak values were lowered, and passive measures, including sound-insulating bore liner, MiniMuff ear pads, and sound-attenuating ear protectors were used (Deoni et al., 2012).

Processing involved linear co-registration of each subject’s raw SPGR, IR-SPGR, and bSSFP images to account for subtle intra-scan motion and removal of non-brain signal. B_0_ and B_1_ field map calibration was followed by MWF map calculation through the iterative fitting of a three-pool tissue model by a constrained fitting approach providing stable estimates (Deoni et al., 2013). Next, individual MWF maps were non-linearly co-registered to a common standardized space. High flip angle T1-weighted SPGR images, acquired as part of mcDESPOT, were used for this alignment (Deoni et al., 2012), and the obtained transformation matrix was applied to individuals’ MWF maps. Maps were then smoothed with a 4 mm full-width-at-half maximum 3D Gaussian kernel within a white and gray matter mask. Finally, mean MWF values were calculated for anatomical masks as defined in Deoni et al. (2012), including whole brain, frontal, parietal, occipital, temporal lobe, cerebellum, cingulum, corona radiata, capsula interna, optic radiation, superior longitudinal fascicle, and corpus callosum. MWF was calculated as the average of left and right hemisphere.

## Results

**FIGURE 3:**
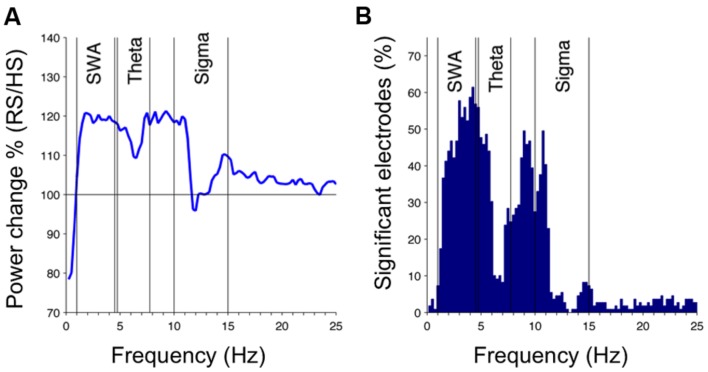
**Homeostatic sleep SWA response to sleep restriction in children: effect of RS (50% reduction of HS) on the sleep EEG in the first 60 min of artifact-free non-REM sleep.** **(A)** Effects of RS on EEG power density spectra presented as RS/HS in percentage, averaged across all 109 channels. **(B)** Percentage of channels reaching significant differences between RS and HS as a function of frequency (paired *t*-test, *df* [mean across 0.25-Hz frequency bins and 109 electrodes] = 23, *p* < 0.05).

**FIGURE 4:**
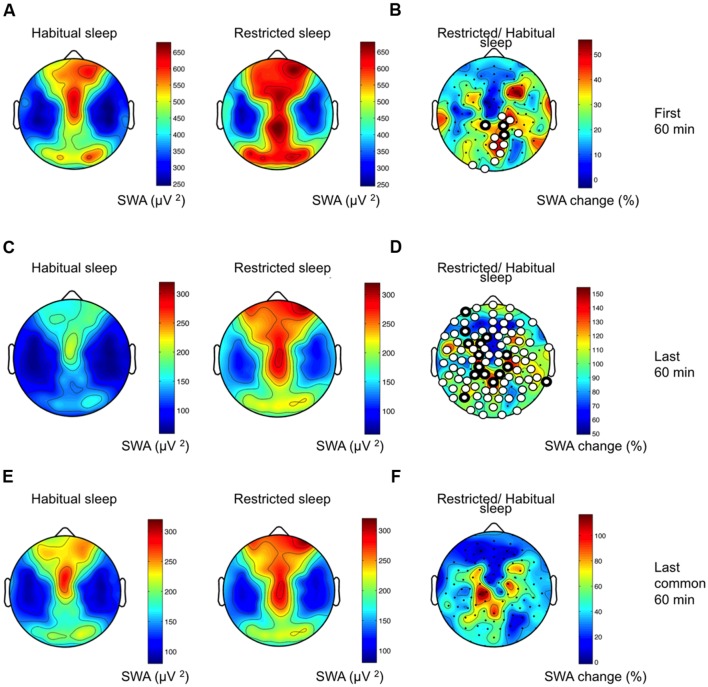
**Local sleep homeostasis.** **(A)** Topographical SWA maps of HS and RS (group average data of 109 channels in both conditions). Values are color coded (maxima in brown, minima in blue) and plotted on the planar projection of a hemispheric scalp model. Values between electrodes were interpolated. **(B)** Percentage change in SWA as RS/HS. Statistical non-parametric mapping (SnPM) of the homeostatic SWA response resulted in a cluster consisting of 13 electrodes exhibiting increased SWA after RS (white circles; critical *t*-value = 2.18). Three electrodes in the cluster survived single-threshold tests (white circles with thicker black boundaries). **(C)** Topographical SWA maps of HS and RS for the last 60 min non-REM sleep. **(D)** Percentage change in SWA as RS/HS for the last 60 min of non-REM sleep. **(E)** Topographical SWA maps of HS and RS for the last common 60 min non-REM sleep (approximately last hour of RS and 4th hour of HS). **(F)** Percentage change in SWA as RS/HS for the last common 60 min of non-REM sleep.

To examine the response to sleep restriction, we contrasted the EEG response as RS/HS ratio. Acute sleep restriction induced a global increase in non-REM sleep EEG power over a broad frequency range (**Figure 3**, first 60 min). The largest increase occurred in the slower frequencies (SWA and frequencies up to 10 Hz) as observed in the increased percentage of EEG power (**Figure 3A**) and in the number of electrodes showing a significant change in power (**Figure 3B**). We also observed an increase in theta and sigma power. This sleep restriction induced effect in sigma power was frequency-specific (increase at 10–11 and 14–15 Hz, and little-to-no between 12 and 13 Hz) and occurred in a local manner (i.e., a high proportion of electrodes changed in the 10–11 Hz range in contrast to a low proportion of electrodes changing in the remaining sigma range). Regionally, the increase in SWA occurred within a parieto-occipital cluster (**Figures 4A,B**; 13 electrodes; three survived the restrictive single-threshold tests). The SWA increase remained when taking into account the last 60 min of artifact-free non-REM sleep; however, this effect was characterized by a global rather than a local response (significant effect in a cluster consisting of 95 electrodes, whereof 15 survive single-threshold test, critical value *t* = 3.17; **Figures 4C,D**). When comparing the last common 60 min (**Figures 4E,F**), no difference between the conditions remained. Thus, the homeostatic SWA response to sleep restriction was most pronounced over posterior regions and showed the strongest regional differentiation in early sleep. In the next steps, we thus capitalized on the first 60 min of non-REM sleep.

**FIGURE 5:**
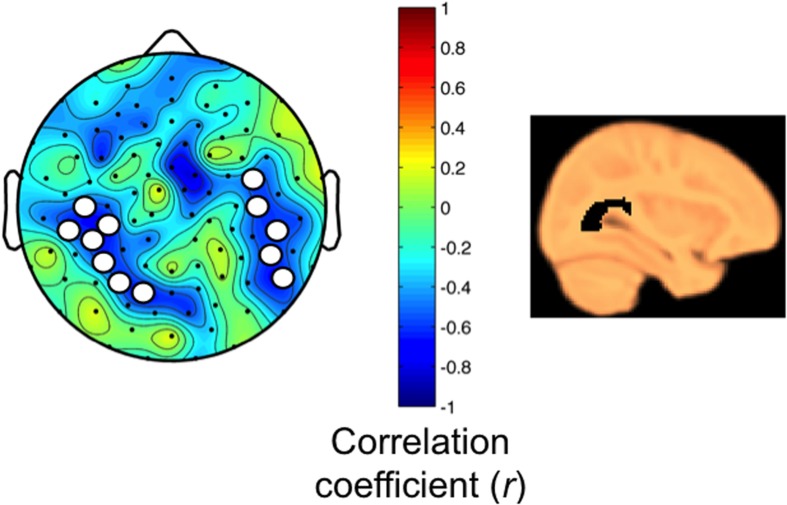
**(Left)** MWF in the optic radiation is related to the homeostatic SWA response (Pearson correlation at each electrode, two-tailed, *p* < 0.05; SnPM corrections for minimal cluster size; correlation coefficients *r* shown in map). Bilaterally, parieto-temporal areas revealed a negative relationship with the homeostatic SWA response (RS/HS, first 60 min non-REM sleep). Electrode clusters consist of 7 and 5 electrodes (cluster size threshold 5). Electrodes within clusters did not survive single threshold tests (critical value *r* = ± 0.84). **(Right)** Sagittal view of optic radiation.

Next, we examined associations between myelin content and the regional SWA response to sleep restriction. As expected for the age group studied (Deoni et al., 2012), correlations between participant age and MWF in the anatomical masks revealed positive relationships (range: 0.45–0.82; all *p* < 0.05, except for the optic radiation *p* = 0.05, one-tailed). Two brain areas showed a negative relationship between the SWA response and MWF content in the optic radiation (**Figure 5**, correlation coefficient *r* ranged from -0.57 to -0.67), which survived controlling for multiple comparisons yet not the single threshold tests. These parieto-temporal areas do not overlap with the region showing the largest SWA rebound after sleep restriction (parieto-occipital cluster first identified in **Figure 4**). No other anatomical masks showed associations between MWF and SWA.

## Discussion

This study examined the effects of acute sleep restriction via a 50% bedtime delay on sleep EEG activity and associations with brain myelin content in 5- to 12-year old children. The main finding shows a local homeostatic SWA response to RS in children that implies high neuronal responsiveness to sleep restriction in a specific parieto-occipital region. Evidence in adults indicates that the homeostatic SWA increase in response to sleep deprivation occurs in anterior as well as posterior derivations (Cajochen et al., 1999; Finelli et al., 2001; Knoblauch et al., 2002). We found a negative relationship between the homeostatic SWA response and MWF in the optic radiation bilaterally, though not entirely symmetrical. These data suggest that sleep need in parieto-temporal areas is related to myelin content. Yet, it is likely that factors other than MWF may determine the immature homeostatic response in school-age children, as the areas with maximal SWA rebound were not related to myelin content in MWF of any of the anatomical masks investigated here. These preliminary and novel findings expand previous knowledge about behavioral, sleep homeostatic and neurocognitive processing responses to sleep loss in children and adolescents (Jenni et al., 2005; Berger et al., 2012; Molfese et al., 2013; Miller et al., 2014).

To our knowledge, this is the first study to show that acute sleep restriction in children induces a neuronal topographical response that differs from adults. While adults exhibit the most pronounced response to RS over prefrontal brain regions (Cajochen et al., 1999; Finelli et al., 2001; Knoblauch et al., 2002), our data show that children‘s response is most pronounced over parieto-occipital areas, suggesting that developing brain circuits are particularly sensitive to RS. Although, our observed effects according to frequency were similar to published data in adults (Finelli et al., 2001), the region-specific power increase in the faster sigma range in our childhood sample is not consistent with findings from older age groups and warrants further investigation.

Myelin growth is a main contributor to overall maturation of brain connectivity (Fields, 2008) and cognitive functions (O’Muircheartaigh et al., 2013), which experience developmental spurts across childhood (Johnson and Munakata, 2005). Recent data indicate a role for sleep in processes associated with membrane synthesis, oligodendrocyte proliferation and myelin growth (Cirelli, 2005; Bellesi et al., 2013). Consistent with these observations, we found a relationship between MWF in the optic radiation and the SWA response; however, this association was not observed in the exact same area where the SWA response was most pronounced (**Figures 4** and **5**). The optic radiation connects the lateral geniculate body and the posterior thalamus with the primary visual cortex of the occipital lobe. Functionally, the optic radiation is a part of visual, spatial-attentional circuits (Toosy et al., 2004) and inter-related with multi-sensory integration (Pasqualotto and Proulx, 2012). Because recent evidence shows that neurodevelopment is more protracted than previously appreciated (Petanjek et al., 2011), prolonged plasticity of multi-sensory circuits may provide fine-tuning of environmental adaptations (Ernst, 2008). Indeed, the optic radiation continues to undergo microstructural changes through childhood (Deoni et al., 2012). Even in our small sample, we found a developmental trend in the increase of MWF in the optic radiation (i.e., correlation with age, *r* = 0.47, *p* = 0.05; one-tailed), which supports the prolonged development of multisensory integration circuits during childhood (Gori et al., 2008). Thus, considering that local sleep SWA reflects cortical plasticity (Steriade and Timofeev, 2003) and parallels the spatial trajectory of neurodevelopment (Kurth et al., 2010), we propose that the posterior homeostatic SWA response may pinpoint brain regions most susceptible to sleep loss.

In adults, individual differences in the white matter volume of the corpus callosum explain 38% of the variability of SWA (Buchmann et al., 2011a). Moreover, as measured with diffusion tensor imaging (DTI), frontal white matter tracts (forceps minor, anterior corpus callosum) and the temporal fascicle are associated with slow wave morphology, such that adults with higher axial diffusivity are more likely to exhibit steeper slow wave slopes (Piantoni et al., 2013). Yet, SWA undergoes remarkable maturation from childhood through adolescence (Campbell and Feinberg, 2009; Kurth et al., 2010), and our findings suggest that the brain response to sleep restriction varies also with age. It is therefore fundamental to understand to what degree brain maturation is *reflected* in the sleep EEG (i.e., experience-independent, developmental changes) and whether brain maturation is *facilitated* by the process of sleep itself (i.e., experience-dependent/experience-expectant).

Electroencephalogram slow waves arise from slow oscillations of the membrane potential that alternate between hyperpolarized “down” states and depolarized “up” states (Steriade et al., 1993a,b). When sleep pressure is high, fluctuations in membrane potentials are highly synchronized across neuronal networks (Vyazovskiy et al., 2009), which gives rise to large amplitude slow waves on the scalp EEG. Cortical activity is also determined by structural and functional properties of connecting subcortical myelinated fibers (Crunelli and Hughes, 2010; Alcauter et al., 2014; O’Muircheartaigh et al., 2015). Relatedly, mechanistic bi-directional interactions between sleep and myelin plasticity have been proposed via oligodendrocyte proliferation, the mediators of myelin formation (Bellesi et al., 2013). Interestingly, oligodendrocyte development and myelination may be closely linked to neuronal activity (Stevens et al., 2002; Fields, 2011). The myelin sheath provides metabolic supplies for high neuronal activity and maintenance (Lee et al., 2012), and neural activity-induced glutamate release along axons seems to trigger myelination (Kukley et al., 2007). During development, both cortical neurons and subcortical myelin are to some degree plastic. Because different brain regions experience periods of increased plasticity at different times during development (Mychasiuk et al., 2013), sleep-wake-plasticity interactions may not be only *age-specific* but also *region-specific*.

Our results represent an important first step in understanding associations between white matter myelin content and local sleep need in the developing human brain. We believe that mapping SWA using high-density sleep EEG is a particularly useful, non-invasive imaging modality for monitoring the brain in pediatric populations, where other functional imaging methods are often difficult or impossible to use (Lustenberger and Huber, 2012). These data are critical for gaining new insights into the possible vulnerability of the developing brain as a result of insufficient sleep; however, our findings need to be interpreted in the context of several limitations. Foremost, the generalizability of our results is reduced because we studied only healthy, good sleeping children whose families agreed to complete the experimental protocol. Further, this is a small, cross-sectional sample in which we used SnPM to reduce the likelihood of type-I errors by controlling for multiple comparisons. Finally, while sleep deprivation in adults impairs cognitive functions related to the prefrontal cortex (Harrison and Horne, 1998; Drummond et al., 1999; Chee and Choo, 2004), whether, sleep deprivation in school-age children similarly impairs functions associated with parieto-occipital brain areas needs further investigation.

In summary, our findings in school-age children revealed a local sleep homeostatic response following acute sleep restriction as measured by increased SWA in parieto-occipital areas and a negative relationship between the homeostatic SWA increase in adjacent, parieto-temporal areas and local myelin content in the optic radiation. Our data suggest high plasticity over parietal-occipital areas in children, which is consistent with anatomical (Kolb and Gibb, 2014), neuroimaging (Giedd et al., 2015) and behavioral data (Spear, 2000; Dumontheil, 2014). In the past, SWA has been linked to cortical plasticity (Huber et al., 2004); however, it has been suggested that cortical activity and subcortical fiber plasticity may trigger reciprocal growth and maintenance (Alcauter et al., 2014). Of note, our data show that the SWA rebound after sleep restriction in children is strongest at the beginning of the night and levels off across time asleep (last common 60 min), which is consistent with existing knowledge of the homeostatic time course of sleep regulation, as reviewed in Achermann and Borbély (2011). In comparison, our finding of a global difference in sleep pressure between experimental conditions in the last 60 min of sleep may provide insights into understanding a pathway by which chronic insufficient sleep and poor behavioral outcomes are linked (Kurth et al., 2015). Sleep deprivation in adults not only enhances SWA but also decreases fast spindle activity, and a reciprocal relationship between these two events has been implied (Aeschbach and Borbely, 1993; Knoblauch et al., 2002). Because we observed the opposite in children – sleep restriction induced mostly an increase in slow sigma power (**Figure 3A**), which was region-specific – the SWA-spindle relationship warrants further analysis.

## Conclusion

We propose that the local SWA response to acute sleep restriction in children signifies increased plasticity related to ongoing neural refinement processes. Our findings also indicate that the local appearance of the electrophysiological marker of sleep homeostasis, SWA, is related to brain myelin morphology. Future studies are needed to investigate the functional consequences of inadequate sleep during different stages of development and to identify the key factors involved in the generation of the posterior homeostatic response in school-age children.

## Author Contributions

SK designed research, performed research, contributed analytic tools, analyzed data, and wrote the paper. DD performed research and analyzed data. PA designed research and wrote the paper. JO performed research and contributed analytic tools. RH contributed analytic tools. SD designed research, performed research, contributed analytic tools, and wrote paper. ML designed research and wrote the paper.

## Conflict of Interest Statement

The authors declare that the research was conducted in the absence of any commercial or financial relationships that could be construed as a potential conflict of interest.
